# Depression and diabetes distress in adults with type 2 diabetes: results from the Australian National Diabetes Audit (ANDA) 2016

**DOI:** 10.1038/s41598-018-26138-5

**Published:** 2018-05-18

**Authors:** Natalie Nanayakkara, Anthony Pease, Sanjeeva Ranasinha, Natalie Wischer, Sofianos Andrikopoulos, Jane Speight, Barbora de Courten, Sophia Zoungas

**Affiliations:** 1 School Public Health and Preventive Medicine, Monash University, Melbourne, VIC 3168 Australia; 2 Diabetes and Vascular Medicine Unit, Monash Health, Clayton, VIC 3168 Australia; 3 National Association Diabetes Centres, Sydney, NSW 2000 Australia; 4 The University of Melbourne, Department of Medicine, Heidelberg, VIC 3168 Australia; 5 Deakin University, School of Psychology, Geelong, Victoria Australia; 6 The Australian Centre for Behavioural Research in Diabetes, Diabetes Victoria, Melbourne, Australia; 7 AHP Research, Hornchurch, UK; 8 The George Institute for Global Health, Camperdown, NSW 2050 Australia

## Abstract

This study explores the prevalence of, and factors associated with, likely depression and diabetes distress in adults with type 2 diabetes in a large, national sample. Australian National Diabetes Audit data were analysed from adults with type 2 diabetes attending 50 diabetes centres. The Brief Case find for Depression and Diabetes Distress Score 17 were administered to screen for likely depression and diabetes-related distress, respectively. A total of 2,552 adults with type 2 diabetes participated: (mean ± SD) age was 63 ± 13 years, diabetes duration was 12 ± 10 years, and HbA1c was 8 ± 2%. Twenty-nine percent of patients had likely depression, 7% had high diabetes distress, and 5% had both. Difficulty following dietary recommendations, smoking, forgetting medications, and diabetes distress were all associated with greater odds of depression whereas higher own health rating was associated with lower odds (all p < 0.02). Female gender, increasing HbA1c, insulin use, difficulty following dietary recommendations and depression were all associated with greater odds of diabetes distress & older age, higher own health rating and monitoring blood glucose levels as recommended were associated with lower odds (all p < 0.04). Depression was associated with sub-optimal self-care, while diabetes distress was associated with higher HbA1c and sub-optimal self-care.

## Introduction

Driven by ageing, obesity and sedentary lifestyles, type 2 diabetes mellitus (T2DM) currently affects just under 400 million individuals worldwide and is expected to rise exponentially, affecting 592 million by 2035^1^. Globally, depression and diabetes represent the 4^th^ and 8^th^ cause of disability adjusted life years respectively^2^. Depression and distress in T2DM are associated with greater morbidity, mortality and healthcare costs^3^.

Like many other chronic conditions, T2DM is associated with depression. Depression has been reported to affect up to 40% of patients with diabetes^4–6^. Indeed, individuals with diabetes have a 2–4 fold greater risk of depression when compared to individuals without diabetes^7^. Depression is associated with higher glycated haemoglobin (HbA1c)^8^, higher rates of complications^9,10^ and mortality^11^. This association may be mediated by sub-optimal self-care^12^, medication adherence^13^, health-related quality of life^14^ and common pathophysiological mechanisms via stress and inflammation^7^. Furthermore, even modest levels of depression are associated with less diabetes self-care^15^. Timely diagnosis and treatment of depression may improve quality of life and increase social and workforce participation for patients as well as family, friends and carers^16,17^.

The negative impact of depression on diabetes may be explained by diabetes distress^18,19^. Diabetes distress refers to the emotional distress related to living with and managing diabetes, not attributable to other causes of overall emotional distress or mental health problems^19,20^. Many adults with diabetes and depressive symptoms experience high levels of emotional distress stemming from their concerns and worries about diabetes^21^.

 Population-based surveys demonstrate that that both depression and diabetes distress are prevalent among adults with type 2 diabetes^22,23^. Despite this, there is limited data exploring factors associated with depression and diabetes distress in Australian people with type 2 diabetes. We hypothesised that significant levels of depression and diabetes distress in adults with T2DM attending diabetes clinics, are related to demographic, self-care, clinical and healthcare factors. Thus, this large-scale, national, cross-sectional clinic-based study examined the prevalence and factors associated with depression and diabetes distress in adults with T2DM attending diabetes centres across Australia.

## Methods

### Participants and procedures

Data were analysed from the Australian National Diabetes Audit (ANDA) including patients from 50 diabetes centres across Australia. The state and territory location of participating centres is presented in Supplementary Table 1. Validated screening instruments were administered during the consultation by clinicians on the day of the survey.

Only adults with T2DM were eligible to be included in the current study; adults and children with type 1 diabetes, women with gestational diabetes and those with other forms of diabetes were excluded.

De-identified data were collected by participating diabetes centres during a single 4-week survey period (May/June 2016). Patients were under the care of endocrinologists, general specialists, general practitioners and diabetes nurse educators. Health professionals from participating centres interviewed patients, reviewed medical records and pathology results before recording the information in a standardised data collection form.

All missing data, invalid entries and discrepancies were clarified with the relevant diabetes centres. The conduct of ANDA and use of de-identified data for research purposes was approved by the Monash Health Human Research Ethics Committee and all research was performed in accordance with relevant guidelines/regulations.

### Explanatory Variables

Pre-specified demographic data (gender, date of birth) and clinical variables (diabetes duration [years], smoking, health professional attendance, glycated haemoglobin A1c [(HbA1c) (within 12 months)], physical activity, diet and medication adherence) were obtained for patients with T2DM. Age and diabetes duration at survey were calculated as year of survey (2016) minus year of birth or year of diabetes diagnosis, respectively. Current smoking status was ascertained at the time of surveys as was physical activity, with sufficient activity defined as ≥150 total minutes per week as per National Physical Activity Guidelines for Australians^24^. Own Health State Rating was determined on a visual analogue scale (0–100) based on patients’ subjective assessment of their health on the day of survey. Patients were considered to be receiving treatment for depression if they were either undergoing counselling by either a psychologist or psychiatrist and/or taking antidepressant medications (not prescribed for peripheral neuropathy).

### Outcome variables

#### Depression

The Brief Case find for Depression (BCD) was administered to screen for likely depression^25^. Depression was considered likely if at least two affirmative answers were indicated, one in each category of the BCD.

#### Diabetes Distress

The Diabetes Distress Score 17 (DDS17) was administered to screen for diabetes-related distress^26^. Patients were first asked two screening questions, if at least one answer was positive, patients were asked to proceed to the DDS17 questionnaire. The DDS17 questionnaire assesses difficulties related to diabetes experienced during the past month, graded on a Likert scale from 1 (not a problem) to 6 (very serious problem). The DDS17 yields a composite score as well as four subscale scores, each exploring a different source of diabetes distress: emotional burden, physician-related distress, regimen-related distress, and interpersonal distress. An overall mean score <2.0 indicates little to no diabetes distress, from 2.0–2.9 indicates moderate diabetes distress, and ≥3.0 indicates a high diabetes distress^27^. Diabetes distress was considered as a dichotomous variable, with patients deemed to have high diabetes distress if DDS17 scores were ≥3.

### Statistical analysis

Categorical variables were summarised as percentages and differences between subgroups analysed using χ^2^ test. Continuous variables were reported as means with standard deviations (SD) or as medians with interquartile ranges (IQR) and tested for normality to determine appropriate statistical analysis (parametric or non-parametric). T tests were performed for normally distributed data and Mann-Whitney U tests for non-normally distributed data. Logistic regression was used to examine factors (age, gender, language, duration, HbA1c, physical activity, diet, smoking, insulin use, medication adherence, glucose monitoring, own health rating and health professional attendance) associated with likely depression and high diabetes distress (as per the categories above). The selection of variables to be entered into each model was based on identifying all variables with possible prognostic importance for the outcomes of interest (and/or exhibiting p < 0.10 on univariate analysis). All analyses were adjusted for age and gender. Patients with missing data for a particular variable were not included in analyses pertaining to that variable, but were not excluded from other analyses where relevant data were available. A two-sided significance level of 0.05 was considered statistically significant. All analyses were performed using Stata software version 14.2 (StataCorp, Texas, USA).

### Data Sharing Statement

Application for datasets generated during and/or analysed during the current study may be considered by the corresponding author on reasonable request.

## Results

Data from 2,552 adults (≥18 years of age) with T2DM were analysed. Mean ( ± SD) age was 63 ± 13 years, T2DM duration was 12 ± 10 years, and HbA1c was 8.0 ± 2.0%. Country of birth was reported as Australia by most patients (65%) followed by England (4%) and New Zealand (3%). No depression and little to moderate diabetes distress was reported by 1663 adults (65%), no depression and high diabetes distress by 56 adults (2%), depression and little to moderate diabetes distress by 578 adults (23%), and depression and high diabetes distress by 120 adults (5%). Participant characteristics are described in Table 1.

**Table 1:** Participant characteristics by depression and diabetes distress status.

Characteristic	Likely depression			
	Yes		No	
N = 2552	Diabetes Distress		Diabetes Distress	
	Yes	No	Yes	No
Participants (n)	126	572	51	1668
Age to 2016 (years), mean (SD)	56.4 ± 11.3	60.5 ± 12.6	54.7 ± 13.0	64.1 ± 12.5
Male, n (%)	51 (41)	306 (54)	19 (37)	954 (57)
Non-English speaking^2^	2 (2)	29 (5)	0 (0)	88 (5)
Diabetes duration (years), mean (SD)	12.2 ± 8.3	11.8 ± 9.6	10.8 ± 7.2	11.7 ± 9.7
**Lifestyle**				
Sufficient Physical Activity^3^, n (%)	19 (15)	176 (31)	15 (29)	705 (42)
Difficulties following the recommended diet, n (%)	91 (72)	280 (49)	36 (71)	525 (31)
Current smoking, n (%)	26 (21)	100 (18)	8 (15.7)	164 (9.8)
**Diabetes management**				
HbA1c^1^ (%), mean (SD)	9.3 ± 2.1	8.4 ± 1.9	9.3 ± 2.0	8.1 ± 1.8
HbA1c^1^ (mmol/mol), mean (SD)	78.4 ± 23	68.1 ± 20.7	78.6 ± 20.8	65.2 ± 20.1
Above target HbA1c (7.0%), n (%)	97 (83)	396 (74)	41 (84)	1057 (68)
Insulin, n (%)	96 (76)	362 (63)	38 (74)	915 (55)
Do you forget to take your medications? n (%)	66 (52)	209 (37)	24 (47)	360 (22)
**Blood glucose testing**				
Tests blood glucose level as often as recommended	58 (46)	159 (28)	19 (37)	407 (25)
Does not check blood glucose level as often as recommended	62 (49)	368 (65)	30 (59)	1,157 (70)
Unsure of recommendation	6 (5)	39 (7)	2 (4)	94 (6)
**Health professional attendances** ^**3**^				
Diabetes specialist review^4^	85 (67)	377 (66)	37 (73)	1,059 (64)
Diabetes educator review^4^	81 (64)	436 (76)	41 (80)	1,201 (72)
Dietitian, n (%)	58 (46)	306 (54)	27 (53)	811 (49)
Podiatrist, n (%)	88 (70)	378 (66)	31 (61)	1,084 (65)
Ophthalmologist and/or Optometrist, n (%)	96 (76)	471 (82)	44 (86)	1,370 (82)
**Physical health**				
Own health state rating (0–100), mean (SD)	43 ± 20	57 ± 21	57 ± 18	70 ± 18
**Psychological health**				
Depression^5^, n (%)	126 (100)	572 (100)	0 (0)	0 (0)
Treated for depression, n (%)	60 (48)	228 (40)	17 (33)	282 (17)
Taking antidepressants^6^, n (%)	54 (43)	189 (33)	15 (29)	249 (15)
Undergoing counselling, n (%)	26 (21)	103 (18)	8 (16)	97 (6)
Diabetes Distress overall score^7^	126 (100)	0 (0)	51 (100)	0 (0)
Emotional distress score^6^, n (%)	120 (95)	89 (16)	44 (86)	69 (4)
Physician- related distress score ^6^, n (%)	50 (40)	9 (2)	19 (37)	7 (0.4)
Regimen- related distress score ^6^, n (%)	110 (87)	78 (14)	44 (86)	57 (3)
Interpersonal distress score^6^, n (%)	83 (66)	37 (6)	27 (53)	29 (2)

^1^Within 6 months of survey; ^2^defined as requirement of interpreter for appointment; ^3^Sufficient physical activity for health benefit is defined as ≥150 total minutes per week; ^4^Attended within the last 12 months; ^5^As indicated by the Brief Case-Find for Depression (BCD); ^6^Not prescribed for peripheral neuropathy; ^7^As indicated by Diabetes Distress 17 Score; *Categorical variables were summarised as percentages.

### Likely Depression

**Figure 1:**
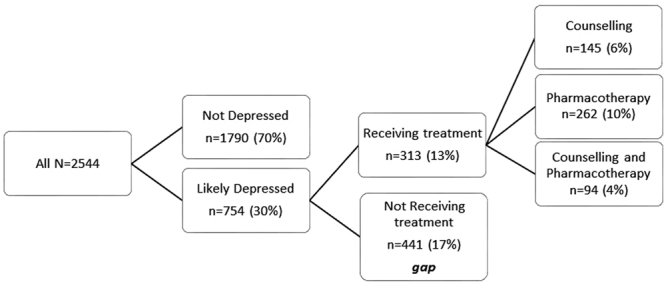
Adults with type 2 diabetes and likely depression.

Thirty percent of adults with T2DM had a BCD score suggesting likely depression, more than half of whom (17% of total) were not receiving any treatment, while just under half (13% of total) were receiving counselling and-/or pharmacotherapy (Fig. 1). Age, female gender, higher HbA1c, insufficient physical activity, difficulty following dietary recommendations, smoking, insulin use, forgetting medications, not monitoring blood glucose levels, treatment for depression, lower own health rating and diabetes distress were significantly associated with depression (univariable p < 0.010, Table 2). In multivariable analysis adjusting for anti-depressant treatment, difficulty following dietary recommendations, current smoking, forgetting medications, and diabetes distress were associated with greater odds of depression whereas higher own health rating was associated with lower odds of depression (all p < 0.002, Table 2). Female gender was not associated with increased risk of depression after adjustment for potential confounding factors such as age, insulin use, HbA1c, smoking status, medication adherence, diet difficulty, physical activity, glucose monitoring, use of antidepressant medications or counselling, own health rating and diabetes distress score [0.96 (0.77–1.18), p = 0.672].

**Table 2:** Factors associated with Likely Depression^#^.

Factor	Univariable Analysis		Multivariable Analysis	
N = 2211	OR (95%CI)	p value	OR (95%CI)	p value
**Age**				
Per 1 year increase	0.97 (0.97–0.98)	<*0*.*001*	0.99 (0.98–1.00)	0.086
**Sex**				
Male (ref)				
Female	1.27 (1.07–1.50)	*0*.*007*	0.96 (0.77–1.18)	0.672
**Non-English speaking** ^**2**^				
Yes (ref)				
No	1.10 (0.74–1.64)	0.638		
**Diabetes Duration**				
1 year increase	1.00 (0.99–1.01)	0.945		
**HbA1c** ^**1**^ **%**				
per unit increase	1.11 (1.06–1.16)	<*0*.*001*	0.99 (0.94–1.05)	0.849
**Sufficient physical activity** ^3^				
No (ref)				
Yes	0.51 (0.42–0.62)	<*0*.*001*	0.84 (0.67–1.05)	0.123
**Difficulties following dietary recommendations**				
No (ref)				
Yes	2.38 (2.00–2.83)	<*0*.*010*	1.42 (1.14–1.78)	*0*.*002*
**Current smoking**				
No (ref)				
Yes	1.97 (1.54–2.51)	<*0*.*001*	1.46 (1.08–1.97)	*0*.*013*
**Insulin use**				
No (ref)				
Yes	1.53 (1.28–1.82)	<*0*.*001*	1.11 (0.88–1.39)	0.376
**Forgets medications**				
No (ref)				
Yes	2.28 (1.90–2.74)	<*0*.*001*	1.47 (1.16–1.85)	*0*.*001*
**Monitors blood glucose as recommended**				
No (ref)				
Yes	0.70 (0.58–0.85)	<*0*.*001*	1.00 (0.79–1.27)	0.998
Unsure of recommended testing	0.90 (0.62–1.31)	0.592	1.22 (0.77–1.94)	0.405
**Treated for depression** ^**4**^				
No (ref)				
Yes	3.34 (2.76–4.03)	<*0*.*001*	2.22 (1.77–2.79)	<*0*.*001*
**Own health rating (1–100)**				
per 1 point increase	0.96 (0.96–0.97)	<*0*.*001*	0.97 (0.97–0.98)	<*0*.*001*
**Diabetes Distress** ^**5**^				
No				
Yes	7.20 (5.13–10.11)	<*0*.*001*	3.18 (2.16–4.69)	<*0*.*001*
**Diabetes Specialist** ^**6**^				
No (ref)				
Yes	1.14 (0.95–1.36)	0.159		
**Diabetes Educator** ^**6**^				
No (ref)				
Yes	1.13 (0.93–1.37)	0.220		

^1^Within 6 months of survey. ^ 2^Defined as requirement of interpreter for appointment. ^3^Sufficient physical activity for health benefit is defined as ≥150 total minutes per week. ^4^Undergoing counselling or prescribed antidepressant medications. ^5^As indicated by Diabetes Distress 17 Score. ^6^Attended within the last 12 months. ^#^As indicated by the Brief Case-Find for Depression (BCD).

### Diabetes Distress

Seven percent of patients had a DDS 17 score suggesting high diabetes distress. Age, female gender, higher HbA1c, insulin use, smoking, forgetting medications, difficulty following dietary recommendations, insufficient physical activity, not monitoring blood glucose levels, depression, treatment for depression and lower own health rating were significantly associated with diabetes distress (univariable p < 0.032 Table 3). In multivariable analysis, female gender, higher HbA1c, insulin use, difficulty following dietary recommendations, and depression were associated with greater odds of diabetes distress whereas older age, higher own health rating and monitoring blood glucose levels as recommended were associated with lower odds of diabetes distress (all p < 0.04, Table 3).

**Table 3:** Factors associated with Diabetes Distress^#^.

Factor	Univariable Analysis		Multivariable Analysis	
N = 2206	OR (95%CI)	p value	OR (95%CI)	p value
**Age**				
Per 1 year increase	0.96 (0.95–0.97)	<*0*.*001*	0.98 (0.96–0.99)	*0*.*006*
**Sex**				
Male (ref)				
Female	1.99 (1.45–2.72)	<*0*.*001*	1.59 (1.11–2.27)	*0*.*012*
**Duration**				
1 year increase	1.00 (0.99–1.02)	0.891		
**HbA1c** ^**1**^ **%**				
per unit increase	1.30 (1.21–1.40)	<*0*.*001*	1.18 (1.07–1.29)	<*0*.*001*
**Insulin use**				
No (ref)				
Yes	2.29 (1.62–3.26)	<*0*.*001*	1.56 (1.02–2.37)	*0*.*039*
**Current smoking**				
No (ref)				
Yes	1.77 (1.19–2.63)	*0*.*005*	0.83 (0.51–1.33)	0.436
**Forgets medications**				
No (ref)				
Yes	3.00 (2.20–4.09)	<*0*.*001*	1.20 (0.82–1.75)	0.339
**Difficulties following dietary recommendations**				
No (ref)				
Yes	4.54 (3.23–6.36)	<*0*.*001*	2.25 (1.52–3.32)	<*0*.*001*
**Sufficient physical activity** ^3^				
No (ref)				
Yes	0.36 (0.25–0.53)	<*0*.*001*	0.72 (0.47–1.12)	0.149
**Monitors blood glucose as recommended**				
No (ref)				
Yes	0.45 (0.33–0.61)	<*0*.*001*	0.67 (0.46–0.98)	*0*.*038*
Unsure of recommended testing	0.44 (0.21–0.93)	*0*.*032*	0.45 (0.18–1.13)	0.091
**Depression** ^**5**^				
No				
Yes	7.20 (5.13–10.11)	<*0*.*001*	3.35 (2.26–4.95)	<*0*.*001*
**Treated for depression** ^**4**^				
No (ref)				
Yes	2.58 (1.89–3.53)	<*0*.*001*	1.21 (0.84–1.77)	0.308
**Own health rating (1–100)**				
per 1 point increase	0.96 (0.95–0.96)	<*0*.*001*	0.97 (0.96–0.98)	<*0*.*001*
**Diabetes Specialist** ^**6**^				
No (ref)				
Yes	1.22 (0.88–1.69)	0.233		
**Diabetes Educator** ^**6**^				
No (ref)				
Yes	0.80 (0.58–1.12)	0.191		

^1^Within 6 months of survey. ^2^Defined as requirement of interpreter for appointment. ^3^Sufficient physical activity for health benefit is defined as ≥150 total minutes per week. ^4^Undergoing counselling or prescribed antidepressant medications. ^5^As indicated by the Brief Case-Find for Depression (BCD). ^6^Attended within the last 12 months. ^#^As indicated by Diabetes Distress 17 Score.

Younger age, insulin use, a requirement for an interpreter, difficulty following dietary recommendations, higher HbA1c, depression, treatment for depression, and lower own health rating were associated with greater odds of emotional distress after adjustment for gender, current smoking, forgetting medications, sufficient physical activity, diabetes specialist review, and not monitoring blood glucose levels (Supplementary Table 2). Younger age, female gender, insufficient physical activity, depression and lower own health rating were associated with greater odds of physician-related distress on adjusted analysis (Supplementary Table 2). Younger age, requirement for an interpreter, forgetting medications, difficulty following dietary recommendations, higher HbA1c, depression, treatment for depression, not monitoring blood glucose levels and lower own health rating were associated with greater odds of regimen-related distress on adjusted analysis (Supplementary Table 2). Younger age, female gender, insulin use, difficulty following dietary recommendations, higher HbA1c, depression, treatment for depression and lower own health rating were associated with greater odds of interpersonal distress on adjusted analysis (Supplementary Table 2). Female gender was associated with increased risk of diabetes distress after adjustment for potential confounding factors such as age, insulin use, HbA1c, smoking status, interpreter use, medication adherence, diet difficulty, physical activity, glucose monitoring, likely depression, use of antidepressant medications or counselling and own health rating [1.59 (1.11–2.27), p = 0.012]. Factors associated with diabetes distress among women and men with type 2 diabetes slightly differed with a greater number among women (younger age, insulin use, higher HbA1c, requirement for interpreter, diet difficulty, being unsure of glucose monitoring recommendations, likely depression and lower own health rating) than among men (higher HbA1c, diet difficulty, likely depression and lower own health rating) (data not shown).

## Discussion

In this large national study, we found that approximately one third of patients with T2DM attending diabetes centres suffer from likely depression and diabetes distress, and that a substantial proportion remain untreated. Patients with depression or diabetes distress were less likely to achieve the recommendations for smoking cessation, diet, physical activity, and blood glucose monitoring. These effects remained significant after adjustment for other relevant confounders.

### Likely Depression

It is of concern that a significant proportion of our patients with T2DM are likely to have comorbid depression with the majority untreated. The rate of depression we observed was comparable to that of other studies from Australia^22^, USA^28^ and China^29,30^. These findings underscore the importance of guidelines recommending clinicians screen, identify and treat depression at the earliest stages of diabetes^31^.

The brief case find for depression is a screening tool for use in general medical and geriatric patients. It has been validated in populations with chronic illness where it was found to have good sensitivity and agreement with other screening tools for depression such as the Primary Care Evaluation of Mental Disorders (PRIME-MD) and the Hospital Anxiety and Depression Scale (HADS)^32,33^. Although clinical psychiatric interview remains the gold standard for diagnosis of depression, this is not usually feasible in the setting of an outpatient diabetes consultation. The BCD can be administered quickly to those at risk of depression before referral to mental health services. A limitation is that it does not allow for determination of symptom severity or impact on daily living and unlike the HADS screen, it does not screen for other mental health conditions such as anxiety. Indeed, depression screening should ideally be followed by further assessment, diagnosis and treatment, if necessary. We did not assess the impact of case finding via the BCD on the subsequent management of patients, and therefore cannot determine in what proportion of patients this screening led to a formal diagnosis of depression.

It is possible that the cross-sectional association we report may be bidirectional. Patients with depression may be more prone to diabetes, or alternatively people with diabetes may be more vulnerable to depression. For example, while some prospective studies have found that depressive symptoms are associated with a 60% increased risk of diabetes^34^ others report a 24% increased risk of depression in patients with diabetes^35^. Further research is required to elucidate the direction and underlying mechanisms linking diabetes^36^ and depression. Some studies suggest that diagnosed diabetes is associated with depression but undiagnosed is not^37^ whereas other studies report that both diagnosed and undiagnosed diabetes are associated with depression, suggesting physiological rather than psychological mechanisms^38^. The limited data examining the relationship between depression and HbA1c levels show mixed results^39^. One study found elevated HbA1c levels among people with diabetes and depression compared with people with diabetes and no depression^40^ whilst other studies found either no relationship between HbA1c levels and depression^41^. Yet others indicate that HbA1c is correlated with depression among people with short but not long term depression^39^. Some studies have also observed a relationship exists between HbA1c levels and depression among people using insulin but not for those using non-insulin hypoglycaemic agents^42^. Furthermore, studies report that pharmacotherapy for the treatment of depression may lead to poorer diabetes control and adverse metabolic indices^43^, although this may vary with the medication used^44^. Understanding these complex relationships may lead to better management strategies and therefore improved outcomes for patients with co-morbid depression and diabetes.

### Diabetes Distress

Diabetes distress needs to be differentiated from depression due to differences in the origins and appropriate management^19^. The 17-item Diabetes Distress Scale (DDS17) is a widely used and validated measure for assessing diabetes-specific distress, with high reliability and validity across many settings, countries and cultures^27,45–47^. The DDS17 items can be used to identify areas of specific patient concern, to enable clinicians to initiate discussions that acknowledge and address diabetes-related difficulties, provide reassurance and initiate behavioural change. Studies indicate that this is most successful when the conversation is initiated by clinicians^48^. Timely detection and management of diabetes distress is associated with better self-care, quality of life and health outcomes^20^. The rate of diabetes distress in our study is comparable to that reported in other studies. We also found that most of the distress reported was in the domains of regimen-related distress and emotional burdens: ‘feeling that they will end up with serious long-term complications regardless of what they do’ and that ‘diabetes and/or hypertension are consuming to much of their mental and physical energy’ followed by ‘feeling that they are not closely adhering to a good meal plan’. In studies conducted in Denmark^49^, China^50^ and Mexico^46^, regimen-related distress and emotional burden were also a greater source of diabetes distress than interpersonal or physician related distress.

### Depression and Distress

Depression and diabetes distress can decrease adherence to self-care practices, and in turn, contribute to higher HbA1c levels^51^. Patients with depression are also less likely to discuss self-care practices with health professionals^52^. Here, we report that HbA1c was associated with diabetes distress, but was not associated with depression after adjustment for other potential confounders. This is likely due to depressive symptoms hindering diabetes self-care, even in patients with symptoms insufficient to make the diagnosis of major depression^36^. We found that diabetes distress but not depression was associated with insulin use after adjustment for other potential confounders. Others^53^ have similarly reported a positive association between diabetes distress and insulin use rather than depression in Turkey and China^30^, suggesting the relationships are consistent across cultures.

### Strengths and Limitations

A strength of this analysis is the nation-wide survey with a large dataset of patients. Data were sourced from the majority of centres registered with the National Association of Diabetes centres (NADC). Thus participants of our study are likely to be representative of patients attending diabetes centres. We obtained information on a broad range of variables with potential impact on mental health. Limitations include that the majority of patients received care at tertiary diabetes centres and may differ from a primary care treated patient group. Further, referral bias is also possible as general practitioners may be more likely to refer more challenging patients whilst managing other patients with better control; skewing results towards a more complex patient cohort with more significant mental health concerns. Alternatively, patients with interrelating co-morbid psychological conditions may also be more likely to be referred. Another limitation was the reliance on self/healthcare worker reports as we were unable to independently verify diagnoses and treatments. This is unlikely to change our findings substantively, as previous research has found approximately 90% of self-reported diabetes information to be valid^54^. We were unable to conduct longitudinal analyses to identify the direction of the reported relationships as the data were obtained in a de-identified format. Our study highlights the need for further prospective studies to examine cause and effect. Our study population was predominantly Australian-born and English speaking, our findings may not be generalisable to other populations. However, the requirement for an interpreter was not associated with either depression or diabetes distress. There was insufficient ethnic variation in our study population for analysis by ethnicity. The BCD is a categorical measure and does not indicate the severity of depression; further studies are required to elucidate if the degree of glycaemic control is associated with the severity of depression. We did not obtain data for the patients who refused or were unable to answer the questions informing this analysis, thus we have no clear indication if or how non-response could have altered our results. However, non-response was rather low (5%).

## Conclusion

The findings of this study emphasise the importance of screening for and addressing emotional and psychological health in people with type 2 diabetes, and highlight the need for longitudinal data to elucidate the determinants of depression and diabetes distress in type 2 diabetes. Given the high prevalence of depression and diabetes distress, routine screening of patients with type 2 diabetes should be encouraged to optimise mental health and improve quality of life.

## Electronic supplementary material

Supplementary Appendix
