# Supplementary material for: Depression and diabetes distress in adults with type 2 diabetes: results from the Australian National Diabetes Audit (ANDA) 2016
Source: Sci Rep. 2018 May 18;8:7846. doi: 10.1038/s41598-018-26138-5 (PMC5959930; doi:10.1038/s41598-018-26138-5)
Supplement: Supplementary file 1 — Supplementary Appendix [file 41598_2018_26138_MOESM1_ESM.pdf]

Supplementary appendix - Depression and diabetes distress in adults with type 2 diabetes: results from the Australian National Diabetes Audit (ANDA) 2016

Natalie Nanayakkara [1,2], Anthony Pease[1,2], Sanjeeva Ranasinha[1], Natalie Wischer[1,3], Sofianos Andrikopoulos[4], Jane Speight[5,6.7], Barbora de Courten[2], Sophia Zoungas[1,2,4]

Affiliations

1. School Public Health and Preventive Medicine, Monash University, VIC 3168, Australia
2. Diabetes and Vascular Medicine Unit, Monash Health, Clayton, VIC, 3168 Australia
3. National Association Diabetes Centres, Sydney, NSW 2000 Australia
4. The University of Melbourne, Department of Medicine, Heidelberg, VIC 3168 Australia
5. Deakin University, School of Psychology, Geelong, Victoria, Australia,
6. The Australian Centre for Behavioural Research in Diabetes, Diabetes Victoria, Melbourne, Australia
7. AHP Research, Hornchurch, UK
8. The George Institute for Global Health, Camperdown, NSW 2050 Australia

Corresponding Author

Professor Sophia Zoungas

School of Public Health and Preventive Medicine, Monash University

Tel.: +61 3 9594 7500; Fax: +61 3 9594 7554

E-mail: sophia.zoungas@monash.edu

Supplementary Appendix

Supplementary Table 1: Participating diabetes centres by state or territory

State/Territory	Participating centres (n)	Adults with T2DM (n)
Australian Capital Territory	1	78
New South Wales	10	480
Queensland	10	699
South Australia	2	111
Tasmania	3	129
Victoria	22	969
Western Australia	2	86
Total	50	2552

Supplementary Table 2 Factors associated with the sub categories of diabetes distress

Multivariable analyses, variables included if p<0.1 on univariable analyses, age and gender always included

Emotional distress	Odds Ratio	[95% Conf.	Interval]	P>z
Age	0.98	0.97	0.99	*0.001*
Sex	1.27	0.96	1.69	0.098
Insulin	1.73	1.23	2.43	*0.001*
Current_Smoking	0.76	0.51	1.13	0.181
Interpret	2.57	1.06	6.20	*0.036*
Medications_Forget	0.97	0.71	1.32	0.837
Diet_Difficulty	2.34	1.73	3.15	*<0.001*
HbA1c Percent	1.14	1.05	1.22	*0.001*
Sufficient_physical_activity	1.12	0.82	1.54	0.482
BCD_depression_likely	3.04	2.26	4.09	*<0.001*
antidep_and/or_counselling	1.43	1.06	1.92	*0.019*
DiabetesSpecialist	1.26	0.91	1.73	0.167
Checks_glucose				
Yes	0.75	0.55	1.02	0.068
Unsure of recommended testing	0.68	0.35	1.32	0.251
Own Health State Rating	0.97	0.96	0.98	*<0.001*

Physician-related distress	Odds Ratio	[95% Conf.	Interval]	P>z
Age	0.98	0.96	0.99	*0.012*
Sex	1.53	0.97	2.41	*0.07*
Insulin	1.46	0.88	2.40	0.141
Medications_Forget	1.44	0.90	2.32	0.131
Diet_Difficulty	1.09	0.67	1.76	0.733
Sufficient_physical_activity	0.53	0.30	0.96	*0.037*
BCD_depression_likely	3.50	2.10	5.85	*<0.001*
Own Health State Rating	0.98	0.97	0.99	*0.004*

Regimen-related distress	Odds Ratio	[95% Conf.	Interval]	P>z
Age	0.98	0.97	0.99	*0.003*
Sex	1.20	0.88	1.63	0.246
Insulin	1.10	0.78	1.53	0.590
Current_Smoking	0.84	0.56	1.26	0.402
Interpret	4.55	1.36	15.18	*0.014*
Medications_Forget	1.70	1.24	2.32	*0.001*
Diet_Difficulty	3.00	2.15	4.17	*<0.001*
HbA1c Percent	1.17	1.09	1.27	*<0.001*
Sufficient_physical_activity	0.77	0.54	1.10	0.148
BCD_depression_likely	2.96	2.15	4.07	*<0.001*
antidep_and/or_counselling	1.57	1.14	2.16	*0.005*
Check_glucose				

Yes	0.39	0.28	0.54	*<0.001*
Unsure of recommended testing	0.59	0.31	1.12	0.108
Own Health State Rating	0.98	0.97	0.98	*<0.001*

Interpersonal distress	Odds Ratio	[95% Conf.	Interval]	P>z
Age	0.98	0.97	1.00	*0.009*
Sex	1.58	1.11	2.26	*0.011*
Insulin	1.72	1.13	2.62	*0.011*
Current_Smoking	0.84	0.52	1.35	0.459
Medications_Forget	1.26	0.87	1.83	0.224
Diet_Difficulty	2.32	1.58	3.40	*<0.001*
HbA1c Percent	1.13	1.03	1.23	*0.007*
BCD_depression_likely	2.93	2.00	4.30	*<0.001*
antidep_and/or_counselling	1.51	1.05	2.17	*0.027*
Check_glucose				
Yes	0.93	0.63	1.36	0.700
Unsure of recommended testing	0.45	0.17	1.21	0.116
Own Health State Rating	0.97	0.96	0.98	*<0.001*

17780

ANDA-AQSMA 2016

Australian National Diabetes Audit - Australian Quality Self Management Audit

nadc National Association of Diabetes Centres

Section 1. Patient Demographics

Medical Record No. ☐☐☐☐☐☐☐☐☐☐ Centre ID ☐☐☐ Site Staff Identifier ☐☐☐☐☐

1.1 Date of birth ☐☐ / ☐☐ / ☐☐☐☐ (d d / m m / y y y y)

1.2 Sex ☐ Male ☐ Female *If FEMALE* → 1.2.1 Currently pregnant ☐ No ☐ Yes

1.3 Date of visit ☐☐ / ☐☐ / 2016 (d d / m m / y y y y)

1.4 Initial visit ☐ No ☐ Yes

1.5 Aboriginal/Torres Strait Islander ☐ No ☐ Yes

1.6 Interpreter required ☐ No ☐ Yes

1.7 DVA patient ☐ No ☐ Yes

1.8 NDSS member ☐ No ☐ Yes

1.9 Country of birth ☐☐☐☐☐☐☐☐☐☐☐☐☐☐☐

Section 2. Diabetes Type & Management & Lifestyle Issues

2.1 Year of diagnosis ☐☐☐☐ (y y y y)

2.2 Type of diabetes ☐ Type 1 ☐ Type 2 ☐ GDM ☐ Don't Know ☐ Other

2.3 Management method ☐ Diet Only ☐ Injectables ☐ Insulin+Tablets+Injectables ☐ Insulin ☐ Tablets ☐ Injectables+ Tablets ☐ Insulin+Tablets ☐ Nil

If INSULIN → 2.3.1 How long ago was insulin started? ☐ <1yr ☐ 1-5yrs ☐ >5yrs

2.4 Physical activity sufficiency ☐ Sufficient ☐ Insufficient ☐ Sedentary

2.5 Have you had a flu vaccination in the last 12 months? ☐ No ☐ Yes

2.6 Have you had a pneumococcal vaccination in the last 12 months? ☐ No ☐ Yes

2.7 Smoking status

☐ Current smoker *If CURRENT* → 2.7.1 Have you tried to stop smoking? ☐ No ☐ Yes

☐ Past smoker *If PAST* → 2.7.2 Which of the following methods did you use?

☐ Never smoked

☐ Just stopped - no intervention ☐ Nicotine replacement ☐ Acupuncture ☐ Medication ☐ Hypnosis ☐ Other

2.8 Glycated Hb result ☐☐ . ☐ % *AND* ☐☐☐ mmol/mol

Section 3. Medication Use

	No	Yes
3.1 Do you ever forget to take your medications?	☐	☐
If YES → 3.1.1 How many times per week? ☐☐		
3.2 Do you usually take all your medications?	☐	☐
3.3 Do you sometimes stop taking your medications when you feel better?	☐	☐
3.4 Do you sometimes stop taking your medications when you feel worse?	☐	☐
3.5 Are you using a complementary therapy or dietary supplement or over the counter (OTC) Rx?	☐	☐
If YES → 3.5.1 Have you told your doctor or educator about using complementary, dietary supplement or OTC Rx?	☐	☐

Section 4. Health Professional Attendances

Has the patient attended any of the following in the last 12 months?

	No	Yes		No	Yes
4.1 Podiatrist	☐	☐	4.6 Diabetes Specialist	☐	☐
4.2 Diabetes Educator	☐	☐	4.7 Ophthalmologist	☐	☐
4.3 Dietitian	☐	☐	4.8 Optometrist	☐	☐
4.4 Psychologist	☐	☐	4.9 Dentist	☐	☐
4.5 Social Worker	☐	☐	4.10 Exercise Physiologist	☐	☐

Section 5. Patient Self Care Practices

	No	Yes
5.1 Do you have difficulties following your recommended diet?	☐	☐
If YES → Do the following apply?		
5.1.1 I don't have enough time to prepare healthy meals	☐	☐
5.1.2 It costs too much to eat well	☐	☐
5.1.3 I don't know what foods are best to	☐	☐
5.1.4 I eat out a lot and find it hard to eat	☐	☐
5.1.5 If Type 1 - it is too hard to count carbs/weigh food	☐	☐

5.2 Do you check your blood glucose level as often as recommended? ☐ No ☐ Yes ☐ Unsure of recommended testing

5.3 If you are on injectables or insulin, do you rotate your injection site? ☐ No ☐ Yes

Section 6A. BCD

Over the last couple of weeks has the patient been:

	No	Yes
6A.1 Having restless or disturbed nights	☐	☐
6A.2 Feeling unhappy or depressed	☐	☐
6A.3 Feeling unable to overcome difficulties	☐	☐
6A.4 Dissatisfied with their way of doing things	☐	☐

Section 6B. Treatment

	No	Yes
6B.1 Is the patient taking antidepressants	☐	☐
6B.2 Psych. treatment/counselling - past	☐	☐
6B.3 Psych. treatment/counselling - now	☐	☐

Section 7. Quality of Life Assessment

Part A: Self-assessment of health status

7.1 Own health state rating (0-100) ☐☐☐

7.2 Screening Scale Q1 ☐

7.3 Screening Scale Q2 ☐

} *If Q1 or Q2 is ≥ 3, complete Part B*

Part B: Diabetes Distress Scale 17

7.4 DDS 17 Questionnaire done ☐ No ☐ Yes

If YES → complete 7.4.1 - 7.4.5 below:

7.4.1 Total DDS 17 Score ☐ . ☐

7.4.2 Emotional Burden (A) ☐ . ☐

7.4.3 Physician-related distress (B) ☐ . ☐

7.4.4 Regimen-related distress (C) ☐ . ☐

7.4.5 Interpersonal distress (D) ☐ . ☐
